# Molecular mechanisms and regulation of recombination frequency and distribution in plants

**DOI:** 10.1007/s00122-024-04590-4

**Published:** 2024-03-21

**Authors:** Meilin Zou, Sergey Shabala, Chenchen Zhao, Meixue Zhou

**Affiliations:** 1 Tasmanian Institute of Agriculture, University of Tasmania, Private Bag 1375, Prospect, TAS 7250 Australia; 2 School of Biological Sciences, University of Western Australia, 35 Stirling Highway, Perth, 6009 Australia

## Abstract

**Key message:**

Recent developments in understanding the distribution and distinctive features of recombination hotspots are reviewed and approaches are proposed to increase recombination frequency in coldspot regions.

**Abstract:**

Recombination events during meiosis provide the foundation and premise for creating new varieties of crops. The frequency of recombination in different genomic regions differs across eukaryote species, with recombination generally occurring more frequently at the ends of chromosomes. In most crop species, recombination is rare in centromeric regions. If a desired gene variant is linked in repulsion with an undesired variant of a second gene in a region with a low recombination rate, obtaining a recombinant plant combining two favorable alleles will be challenging. Traditional crop breeding involves combining desirable genes from parental plants into offspring. Therefore, understanding the mechanisms of recombination and factors affecting the occurrence of meiotic recombination is important for crop breeding. Here, we review chromosome recombination types, recombination mechanisms, genes and proteins involved in the meiotic recombination process, recombination hotspots and their regulation systems and discuss how to increase recombination frequency in recombination coldspot regions.

## Introduction

Meiosis is an essential part of sexual reproduction in most organisms. This process halves chromosome numbers by coupling a single round of DNA replication with two consecutive rounds of nuclear division (meiosis I and meiosis II) to produce haploid gametes (Hillers et al. 2017; Kleckner 1996). During meiosis I, replicated homologous chromosomes align and undergo recombination between non-sister chromatids before separating. Then, during meiosis II, sister chromatids segregate, ultimately producing four gametes (Kleckner 1996) with each gamete containing one set of chromosomes. When one gamete from one sex combines with a gamete of the opposite sex, the chromosome number of the subsequent generation returns to the parental level, maintaining a steady state (Hillers et al. 2017). This process leads to new combinations of alleles present in the progenies. As a result, chromosome recombination during meiosis is regarded as the foundation for genetic diversity and the evolution of species.

Recombination rates vary across different regions of chromosomes (Blair et al. 2018; Henderson 2012; Kauppi et al. 2004). In most crop species, recombination rates are positively correlated with the distance from the centromere and gene densities but negatively correlated with transposable elements (Barakate et al. 2014; Blair et al. 2018; Henderson 2012; Kauppi et al. 2004; Phillips et al. 2010; Shen et al. 2017). The genomic region that has a relatively higher recombination frequency is referred to as recombination hotspot, while the region with a lower recombinant frequency is called a coldspot. Traditional crop breeding relies heavily on incorporating beneficial gene alleles from parental chromosomes into their offspring. Consequently, low recombination frequency hampers the selection of lines that pyramid favorable close-linked traits within coldspots in crop breeding programs. Due to increased food demand and rapidly deteriorating climate change, it is an urgent requirement to create new varieties that would possess high yields and better quality while being climate resilient. However, previous studies indicated that many genes, such as around one-third in barley, are located in recombination coldspot regions (Higgins et al. 2014). This poses a challenge for crop breeders, as achieving their objectives leads to a significant increase in breeding costs and cycle times.

Although recombination mechanisms are not yet fully understood, substantial progress has been made in studying recombination models. This review provides an overview of gene recombination types and genetic mechanisms, the genes participating in the recombination process, the classification of recombination pathways, the distribution of recombination events and their regulatory systems in plants. Additionally, we explore the potential for enhancing recombination rates in coldspot regions.

## Gene recombination and genetic mechanisms

### Recombination types

**Fig. 1:**
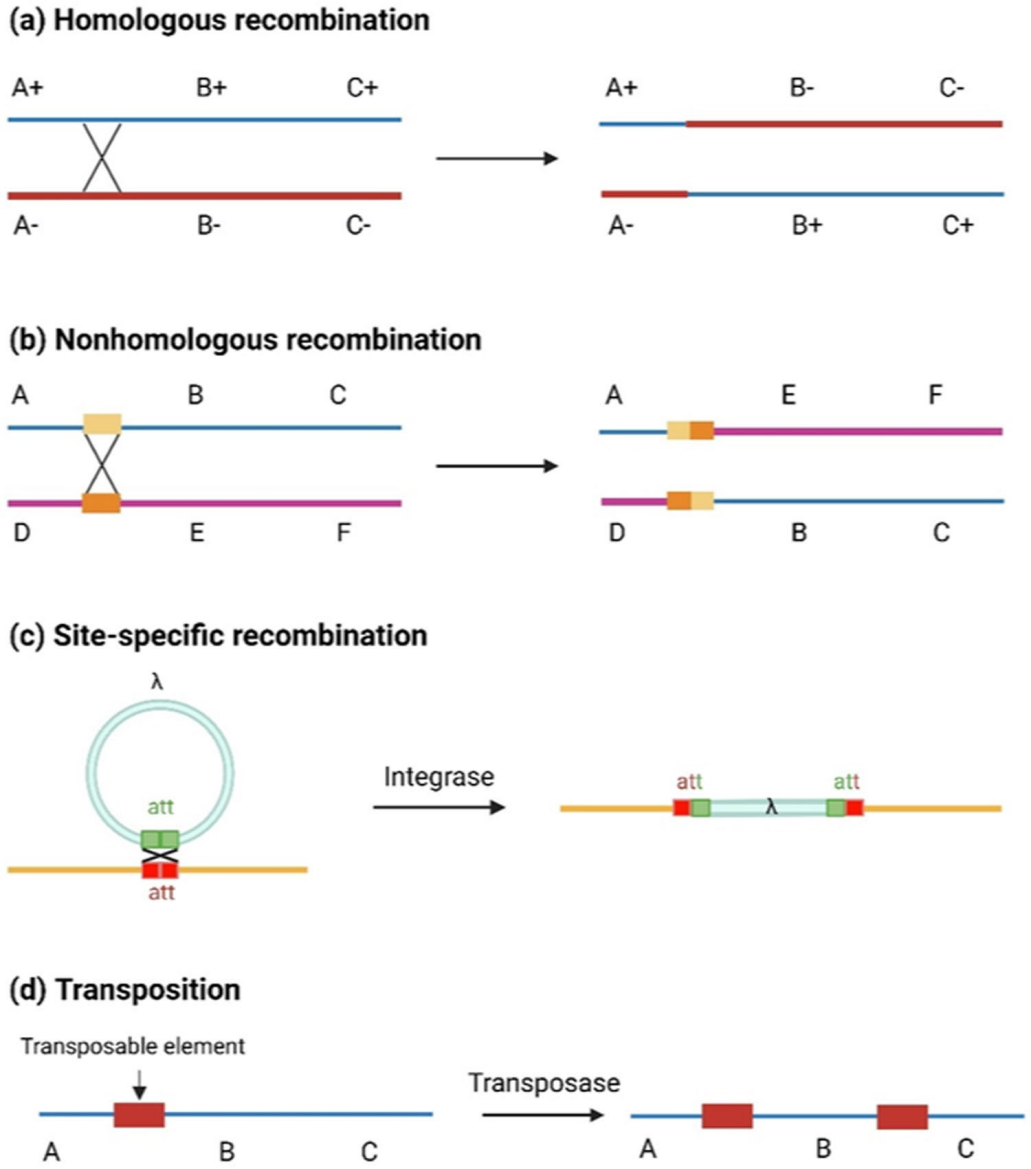
Four recombination types. **a** The process of homologous recombination involves the DNA double-strand break and rejoining the strands. It results in an exchange of genetic information between the homologous chromosomes. **b** The yellow and orange blocks represent the segments at the sites of nonhomologous recombination. It does not require sequence homology between the DNA molecules and can introduce mutations at the site of the break. **c** Site-specific recombination catalyzed by site-specific recombinase enzymes, is usually observed between two different DNA molecules among bacteriophages, bacteria, and unicellular eukaryotes. **d** The red block represents the transposable element of DNA which could be integrated into the genome

In genetics, recombination refers to the process of rearranging genetic material from different chromosomes or regions to create new DNA combinations (Rice 2002; Stapley et al. 2017). It can occur naturally in both eukaryotes and prokaryotes and can also be induced in a laboratory (Baker et al. 1976; Camerini-Otero and Hsieh 1995; Covo et al. 2012; Gratia 2017; Paques and Haber 1999; Schnable et al. 1998). Recombination types are primarily classified into four groups: homologous recombination, non-homologous recombination, site-specific recombination and transposition (Fig. 1).

Homologous recombination, known as general recombination, typically occurs during meiosis in eukaryotic cells (Camerini-Otero and Hsieh 1995). It involves the exchange of genetic information between the alleles of homologous chromosomes and generates genetic diversity in offspring. The occurrence of homologous recombination requires homologous segments that have large-scale significant similar sequences to line up in proximity. Non-homologous recombination process ligates the broken ends of DNA together directly with no requirement of a homologous sequence to serve as a template to repair DNA double-stranded breaks (DSBs) (Pannunzio et al. 2018). It is more prone to mistakes and can give rise to the deletion or insertion of genetic material at the break site and even chromosomal abnormalities. These outcomes can have significant consequences for gene function and regulation (Pannunzio et al. 2018). Site-specific recombination process is catalyzed by site-specific recombinases and reintegrates the DNA segments at specific sites (Grindley et al. 2006). The transposition process involves the movement of transposable elements within the genome. Replicative transpositions may result in the creation of a new copy (Fedoroff 2000). A homologous recombination during meiosis is the primary type of recombination employed in crop breeding programs. Therefore, our focus will be on this specific type of recombination.

### Homologous recombination process

**Fig. 2:**
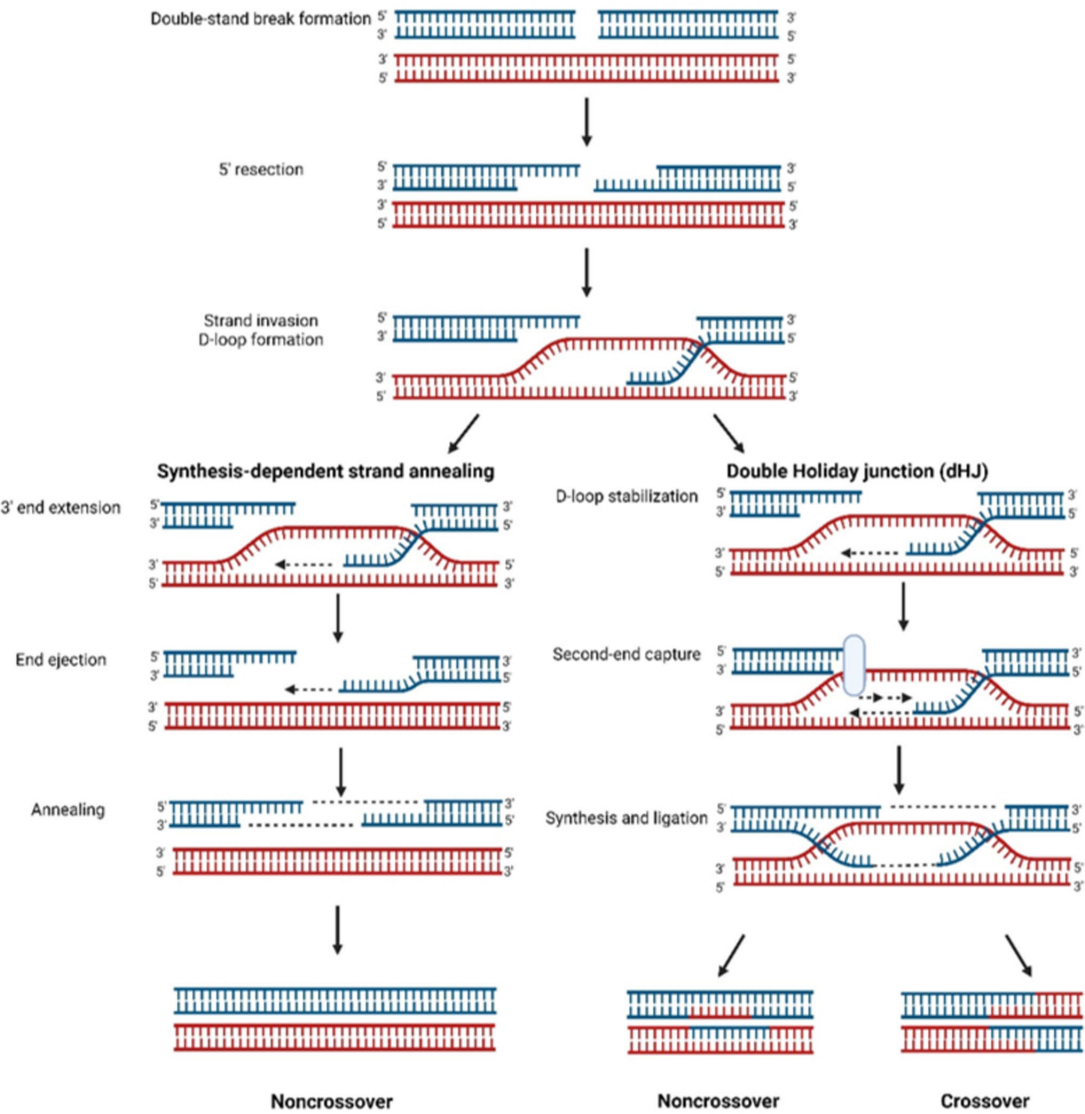
CO and NCO produced from the homologous recombination. The homologous recombination begins with the DNA double-strand breaks of one of the homologous DNA duplexes, shown as blue strands. The 5′ end of the DSB is resected by specific nucleases to generate 3′ single-stranded DNA. One of the 3′ ends invades another homologous DNA duplexes which are shown as red strands, forming a displacement loop (D-loop) structure. DNA polymerase extends the invading 3′ end strand to generate the new DNA. For SDSA, the newly synthesized strand is displaced from the D-loop and then anneals, and typically produces NCO products. The formation of dHJ is derived from capturing the second end of the break after DNA synthesis extends the invading strand. It is resolved by cutting the noncrossed strands and producing NCO or by cutting the crossed strands and creating COs

The genetic recombination process has a variety of forms and presents great complexities, varying among species. In the meiosis of eukaryotes, the widely accepted model of homologous recombination is primarily based on studies of DSB repair in *Saccharomyces cerevisiae* (Aylon and Kupiec 2004; Osman et al. 2011). This process is initiated by programmed DSB and involves rejoining of DNA sequences (Lake and Hawley 2016; Murakami and Keeney 2008). DSB repair could generate either crossover (CO) recombination or non-crossover (NCO) recombination through different pathways, including double Holliday junction (dHJ) model and synthesis-dependent strand annealing (SDSA) model. The CO recombination modifies two chromatids by exchanging large DNA fragments, while NCO only involves copying and replacing a short stretch of DNA without exchange (Fig. 2). Homologous chromosome pairing and recombination occur in the prophase phase of meiosis I (prophase I) (Azumi et al. 2002; Zickler and Kleckner 2015). The chromosomes start to condense and become thin filaments that could be visible under the light microscope during the Prophase I leptotene stage (Hartl and Ruvolo 2012). At the zygotene stage, homologous chromosomes align closely through the formation of a synaptonemal complex (SC), a unique proteinaceous structure (Fraune et al. 2012; Hartl and Ruvolo 2012; Heyting 1996; Hillers et al. 2017; Page and Hawley 2004). The SC is completely assembled during pachytene stage, and is considered to promote the initiation of recombination events (Fraune et al. 2012; Hartl and Ruvolo 2012; Hayashi et al. 2010; Hernandez-Hernandez et al. 2016; Hillers et al. 2017; Kouznetsova et al. 2011; Schramm et al. 2011). The CO takes place between two non-sister chromatids of the homologous chromosomes during the pachytene stage (Gilbert and Barresi 2016; Hartl and Ruvolo 2012). In the diplotene stage, homologous chromosomes start to separate from each other with the dissolution of SC and are only attached at chiasmata (Armstrong and Jones 2003; Hartl and Ruvolo 2012; Heyting 1996). Finally, the chromosomes become fully condensed during the diakinesis stage (Hartl and Ruvolo 2012; Taiz et al. 2015). Some studies suggest that homology along the chromosome arms is the main determinant of the recognition and pairing of homologous chromosomes, with centromeres playing a negligible role in this process during meiosis (Corredor et al. 2007; Lefrancois et al. 2016).

### Genes involved in DSB formation

DSB could be caused by exogenous or endogenous factors in a variety of circumstances. Genes that function in DSB formation are exceptionally diverse, which makes the study of the mechanism extremely complex (Table 1). During meiosis, the conserved SPO11 protein is one of the primary participants involved directly in the DSB process (Keeney and Neale 2006; Lam and Keeney 2015). It shares homology with the subunit A of archaeal topoisomerase VI (TopVIA), a type II DNA topoisomerase (Bergerat et al. 1997; Gadelle et al. 2003; Keeney 2008). Two hybrid active sites of the Spo11 contain tyrosine which reacts with the phosphodiester linkage of DNA to cleave DNA strands (Diaz et al. 2002; Nichols et al. 1999; Shingu et al. 2010). In addition, the homolog of archaeal topoisomerase VI subunit B (TopVIB), known as the meiotic topoisomerase VI B subunit (MTOPVIB), forms a complex with SPO11 and is also required for DSB formation in the meiotic recombination process (An et al. 2011; Fu et al. 2016; Robert et al. 2016; Tang et al. 2017; Vrielynck et al. 2016; Xue et al. 2016). Studies in *S. cerevisiae* indicate that SPO11 alone is insufficient to generate DSB. There are at least nine other proteins that promote DSB formation, namely Ski8, Mei4, Mer2, Mre11, Rad50, Rec102, Rec104, Rec114, and Xrs2 (Cole et al. 2010; Lam and Keeney 2015; Neale et al. 2005). They can form several different interacting subcomplexes, Spo11-Ski8, Rec102-Rec104, Rec114-Mei4-Mer2 and Mre11-Rad50-Xrs2 (MRX) (Lam and Keeney 2015; Li et al. 2006; Maleki et al. 2007). It is worth noting that the MRX complex is not only recruited during DSB formation but also plays a role in the subsequent DSB repair processes (Borde et al. 2004; Williams et al. 2007).

**Table 1:** List of genes involved in DSB and repair

Function	Protein	Species	Reference
DSB formation	SPO11-1	*Arabidopsis*	Grelon et al. (2001); Hartung et al. (2007)
		Rice	Yu et al. (2010)
	SPO11-2	*Arabidopsis*	Hartung et al. (2007); Stacey et al. (2006)
	SPO11-4	Rice	An et al. (2011)
	MTOPVIB	*Arabidopsis*	Vrielynck et al. (2016)
		Rice	Xue et al. (2016)
	PRD1	*Arabidopsis*	De Muyt et al. (2007)
	PRD2	*Arabidopsis*	De Muyt et al. (2009)
	PRD3	*Arabidopsis*	De Muyt et al. (2009)
	PAIR1	Rice	Nonomura et al. (2004)
	DFO	*Arabidopsis*	Zhang et al. (2012)
	CRC1	Rice	Miao et al. (2013)
	PHS1	*Arabidopsis*	Ronceret et al. (2009)
		Maize	Pawlowski et al. (2004)
DSB repair	MRE11	*Arabidopsis*	Puizina et al. (2004)
		Rice	Ji et al. (2013)
	RAD50	*Arabidopsis*	Bleuyard et al. (2004)
	NBS1	*Arabidopsis*	Waterworth et al. (2007)
	COM1	*Arabidopsis*	Uanschou et al. (2007)
		Rice	Ji et al. (2012)
	RAD51	*Arabidopsis*	Da Ines et al. (2012); Su et al. (2017)
		Rice	Byun and Kim (2014); Kou et al. (2012); Tang et al. (2014)
		Maize	Li et al. (2007)
	DMC1	*Arabidopsis*	Couteau et al. (1999); Da Ines et al. (2012)
		Rice	Deng and Wang (2007)
	RPA	*Arabidopsis*	Aklilu et al. (2014)
		Rice	Chang et al. (2009b); Li et al. (2013); Shultz et al. (2007)
	BRCA2	*Arabidopsis*	Siaud et al. (2004)
	MND1	*Arabidopsis*	Panoli et al. (2006); Vignard et al. (2007)
	HOP2	*Arabidopsis*	Uanschou et al. (2013)
	XRCC2	*Arabidopsis*	Wang et al. (2014)
	XRCC3	*Arabidopsis*	Bleuyard and White (2004); Su et al. (2017)
	RFC1	*Arabidopsis*	Liu et al. (2013)
	SDS	*Arabidopsis*	Azumi et al. (2002)
		Rice	Chang et al. (2009a)
CO and NCO formation	MER3/RCK	*Arabidopsis*	Chen et al. (2005); Mercier et al. (2005)
		Rice	Chang et al. (2009a)
	MUS81	*Arabidopsis*	Hartung et al. (2006); Higgins et al. (2008a)
	MSH4	*Arabidopsis*	Higgins et al. (2004)
	MSH5	*Arabidopsis*	Higgins et al. (2008b)
		Rice	Luo et al. (2013)
	ZIP4	*Arabidopsis*	Kuromori et al. (2008)
		Rice	Shen et al. (2012)
	PSS1	*Arabidopsis*	Duroc et al. (2014)
		Rice	Zhou et al. (2011)
	Ph1	Wheat	Griffiths et al. (2006)
	HEI10	*Arabidopsis*	Chelysheva et al. (2012)
		Rice	Wang et al. (2012)
	MLH1	*Arabidopsis*	Dion et al. (2007)
	MLH3	*Arabidopsis*	Jackson et al. (2006)
	ZYP1	*Arabidopsis*	Higgins et al. (2005)
		Barley	Barakate et al. (2014)
	FANCM	*Arabidopsis*	Crismani et al. (2012); Knoll et al. (2012)
		Brassica	Blary et al. (2020)
	RTEL1	Barley	Barakate et al. (2021)
	RECQ4	*Arabidopsis*	Seguela-Arnaud et al. (2015)
		Tomato	De Maagd et al. (2020)
	Top3 α	*Arabidopsis*	Seguela-Arnaud et al. (2017); Seguela-Arnaud et al. (2015)
	FIGL1	*Arabidopsis*	Fernandes et al. (2018a)

There are functional divergences and significant sequence evolutionary divergences among some DSB proteins across different species. For example, AtMRE11 and AtRAD50 only participate in the DNA repair process rather than generating DSB**,** and Ski8 is involved in DSB formation in *S. cerevisiae* but not in *Arabidopsis* (Jolivet et al. 2006; Osman et al. 2011). In rice, OsSPO11-1 and OsSPO11-4 participate in DSB formation, while OsSPO11-2 and OsSPO11-3 are not involved in DSB formation (An et al. 2011; Yu et al. 2010). *Arabidopsis* is widely accepted as a model system in plant scientific research, containing three SPO11 homologs, AtSPO11-1, AtSPO11-2, and AtSPO11-3 (Hartung and Puchta 2000; Stacey et al. 2006). However, only AtSPO11-1 and AtSPO11-2 are essential for DSB formation, likely acting as heterodimers in meiotic recombination (Grelon et al. 2001; Hartung et al. 2007; Stacey et al. 2006). Numerous proteins, such as AtPRD1, AtPRD2, AtPRD3 and AtDFO, participate in DSB formation in *Arabidopsis* (Muyt et al. 2009, 2007; Zhang et al. 2012). The protein AtPRD1 shows interactions with AtSPO11-1, AtSPO11-2, MTOPVIB, AtPRD3 and AtDFO, although there is currently no evidence of its interaction with AtPRD2 (Muyt et al. 2007; Tang et al. 2017).

### DSB end processing and a single strand DNA invasion

After DSB, SPO11 remains covalently linked to the 5′ terminal of each broken DNA strand (Keeney et al. 1997; Lam and Keeney 2015; Neale et al. 2005). The MRX complex (composed of MRE11, RAD50, Xrs2) works with Com1/Sae2 (Table 1) to resect the 5′ end on each side of the DSB and remove SPO11 (Aylon and Kupiec 2004; Cannavo and Cejka 2014). Further resections of the 5′ termini are conducted by exonuclease 1 (EXO1), resulting in the generation of 3′ ssDNA tails (Garcia et al. 2011). With the assistance of recombinases, these ssDNA tails invade the homologous duplex DNA to form a recombination intermediate known as D-loop (Hunter 2015; Hunter and Kleckner 2001; Lichten 2001; Martinez-Perez and Colaiacovo 2009; Wang and Copenhaver 2018). A replication protein A (RPA) binds the 3′ terminus to prevent degradation and remove secondary structures, facilitating the recruitment of recombinases (Soustelle et al. 2002; Wold 1997). RPA is a heterotrimeric complex that consists of three subunits: RPA1, RPA2 and RPA3 (Ribeiro et al. 2016) (Table 1). RPA also act on DNA annealing which is promoted by Rad52 protein during second-end capture (Nimonkar et al. 2009; Sugiyama et al. 2006; Wang and Haber 2004). Once the 3′ ssDNA tails are protected, recombinase A (RecA)-like related recombinases are loaded to form presynaptic nucleoprotein filaments. These filaments facilitate the invasion of free 3′ end ssDNA into the duplex DNA of the paired homologous chromosome, forming the D-Loop in yeast (Brown and Bishop 2015; Shinohara et al. 1992). Two homologs of the bacterial RecA, Rad51 and Dmc1, have been discovered in most eukaryotic organisms (Table 1) (Bishop et al. 1992; Brown and Bishop 2015; Shinohara et al. 1992). Both recombinases play crucial roles in efficient meiotic recombination. Rad51 not only directly facilitates recombination during mitosis but also participates in meiotic recombination, whereas Dmc1 is merely required for meiotic recombination (Bishop 2012; Bishop et al. 1992; Shinohara et al. 1992).

### DSB repair

The current meiotic DSB repair model is broadly divided into two categories: the dHJ model and the SDSA model. Both of these models have been observed in yeast system (Mitchel et al. 2010). In the dHJ model, the invading 3′ end function as a primer to initiate the DNA synthesis using invaded DNA as the template (Szostak et al. 1983). Consequently, the newly synthesized DNA contains a specified sequence which is the same as the invaded DNA. The strand invasion and second end capture lead to the formation of dHJ, which are resolved to form CO or NCO products (Szostak et al. 1983; Wyatt and West 2014). The characteristic of the SDSA model is strand displacement, where the invading strand can anneal with the other 3′ single-stranded end (Szostak et al. 1983). In the SDSA model, only one DNA terminal participates in the invasion process, while another one utilizes newly synthesized DNA as a template for synthesis. This process results in the formation of NCO products (Szostak et al. 1983).

In general, the majority of CO products are formed through the dHJ intermediate, while most NCO is primarily produced via the SDSA (Allers and Lichten 2001; McMahill et al. 2007). These intermediates can either undergo a repair, resulting in gene conversions, or they can segregate during the next round of replication. In addition, the dissolution of dHJs could also give rise to some NCO products (Wyatt and West 2014). Usually, only a small proportion of meiotic DSBs are repaired into COs in plants. Meiotic DSBs are generated in excess with more than 90% of plant DSBs being repaired using the sister chromatid as a template or being resolved as NCO (Mercier et al. 2015).

## DSB and CO events are associated with chromosome number and size

The identification of recombination distribution provides valuable insights into genome evolution and plant breeding. Distribution patterns of DSB and CO, along with their hotspots, have been reported in various species (Table 2).

**Table 2:** Recombination profile in different species

Species	DSB	NCO	CO	Chr. pairs	Genome size	Reference
*Arabidopsis*	–	–	8–13	5	0.135 Gb	Lian et al. (2022)
*Arabidopsis*	~ 200	–	7–11	5	0.135 Gb	Sanchez-Moran et al. (2002)
*Arabidopsis*	–	6	9	5	0.135 Gb	Lu et al. (2012)
Maize	–	–	16–19	10	2.4 Gb	Sidhu et al. (2015)
Maize	~ 500	–	20	10	2.4 Gb	Anderson et al. (2003); Pawlowski et al. (2003)
Maize	218–608	–	11.2–19.4	10	2.4 Gb	Sidhu et al. (2015)
wheat	~ 2100	–	55	21	17 Gb	Gardiner et al. (2019)
Barley	–	–	19–24	7	5.1 Gb	Phillips et al. (2015)
Soybean	–	25	49–59	20	1.1 Gb	Ma et al. (2023)
Cucumber	–	–	12.9–13.8	7	0.367 Gb	Wang et al. (2023)

DSB varies considerably across different species. About 200 DSB events in *Arabidopsis* (Sanchez-Moran et al. 2007) and ~ 500 DSBs in maize (Pawlowski et al. 2003) have been detected. More DSB events (2100) have been identified in bread wheat as wheat has a larger genome size and a greater number of chromosomes (Gardiner et al. 2019). This suggests a correlation between the number of chromosomes and the genome size with the frequency of DSB events.

Only a small amount of DSBs are repaired into CO, for example, about 4% in maize (Sidhu et al. 2015). In *Arabidopsis* (2*n* = 10), the number of COs ranged from 7 to 13 across different studies, with six NCOs reported. In soybean (2*n* = 40), the average number of COs per recombinant line is 49–59, while NCOs are about 25, about half of the CO events. In cucumber (2*n* = 14), a dicot species, the average number of COs per individual ranges from 12.9 to 13.8, approximately one for each chromosome. In maize (2*n* = 20), a monocot species, approximately 500 DSBs were identified per cell, while only 20 COs were formed. In bread wheat, 55 COs were identified, while in barley (2*n* = 14), 19–24 COs were estimated across 45 genetic mapping populations (Table 2). The total number of COs is associated with the number of chromosomes. Soybean and bread wheat contain similar pairs of chromosome numbers, 20 and 21, respectively, and their COs are both around 55 (Table 2). For the species with a smaller number of chromosomes (5—10 pairs), e.g. *Arabidopsis*, cucumber, maize and barley, their corresponding COs are less than 25, confirming that the number of COs is positively correlated with the number of chromosomes. Besides, chromosome size also shows influence on CO events. For example, both cucumber and barley have seven pairs of chromosomes, but the genome size in barley is 13.5 times greater than that of cucumber. The considerably longer chromosomes in barley compared to cucumber lead to nearly twice as many.

In plants, typically one to two COs are distributed across most individual chromosomes (Jones and Franklin 2006; Sidhu et al. 2015), with the majority of DSBs being repaired as NCOs through DNA synthesis, utilizing the homologous chromosome as a template or the sister chromatid (Allers and Lichten 2001). Nevertheless, regardless of the mechanisms, as indicated by the studies listed in Table 2, the majority of genetic variation generated by meiotic recombination in plants originates from COs.

The factors determining whether COs or NCOs form from DSB are poorly understood. Studies conducted in mouse, *C.* *elegans,* and budding yeast have indicated that CO numbers are not impacted by DSB numbers, even when the number of DSB varies significantly (Cole et al. 2012; Martini et al. 2006; Rosu et al. 2011; Yokoo et al. 2012). In contrast, a strong correlation between the number of meiotic DSBs and COs has been found in maize (Sidhu et al. 2015) with 25.8% of bivalents having single chiasma, 72.6% forming two chiasmata and only 1.7% displaying three chiasma (Sidhu et al. 2015).

Furthermore, variations in recombination frequency exist between genders within the same species (Lenormand and Dutheil 2005; Martinez-Perez and Colaiacovo 2009). For example, in *Arabidopsis,* the recombination rate is higher males than in females, particularly in the sub-telomeric region (Giraut et al. 2011). A similar difference has been found in barley, with male gametes showing more COs than female gametes (Phillips et al. 2015).

## CO hotspot distribution across chromosomes

A prerequisite for the formation of recombination is the occurrence of DSB. In the search of DSB hotspots, researchers have found a close relationship between the number of hotspots and chromosome length, with the average hotspot length being 1 ~ 2 kb (He et al. 2017; Paul et al. 2016). In maize, there is a low frequency of CO hotspots occur in the centromeric and pericentromeric chromosome regions, while these regions exhibit a high frequency of the DSB hotspots (He et al. 2017). In *S. cerevisiae*, hypomorphic mutants of *spo11* show a decrease in DSB number but not CO numbers (Martini et al. 2006). As the repair of DSB can proceed via either the CO pathway or the NCO pathway, an increase in CO may occur at the cost of NCOs, maintaining homeostasis (Martini et al. 2006). Consequently, there isn’t an absolute correlation between the *spo11* alleles and COs. Therefore, it is impossible to solely identify the recombination spots based on DSB spots.

Meiotic recombination events are unevenly distributed and are restricted to certain regions, particularly at the distal ends of chromosome arms (He et al. 2017; Lukaszewski 1992; Paigen and Petkov 2010; Petes 2001). The preferential distribution of CO hotspots is in gene-rich regions where the chromatin is easily accessible by DSB complex. Furthermore, structure variations, such as large inversions, have been reported to influence the recombination rate in barley. For example, no recombination event can be identified within a large 141 Mb inversion region on chromosome 7H from the DH population of RGT Planet and Hindmarsh (Jayakodi et al. 2020).

Some studies show that recombination hotspot tends to present near gene promoters (Choi et al. 2013; Mancera et al. 2008; Pan et al. 2011; Petes 2001; Wu and Lichten 1994). Research on the hexaploid wheat genome has shown that recombination hotspots typically occur near the coding regions of the chromosomes (Darrier et al. 2017) with about 95% of the recombination being distributed in 18 major and 30 minor gene-rich regions (Erayman et al. 2004). Similarly, studies in maize have suggested that approximately 90% hotspots are distributed in gene-rich regions (Fu et al. 2001; Gore et al. 2009; Kianian et al. 2018; Li et al. 2015; Rodgers-Melnick et al. 2015; Sidhu et al. 2015). A recent comprehensive study in cucumber revealed that over 93% of the COs are either in genes or their 10 kb regions. Among these, about 45% of COs occurred in distal intergenic regions, 25% in the promoter regions (2 kb upstream), 13% in introns, 10% in coding sequences, and 7% in untranslated regions (Wang et al. 2023).

McConaughy et al. (2023) identified 451 CO hotspots from two soybean mapping populations. These hotspots are distributed across the entire soybean genome with around 27% of them located in the pericentromeric regions. In barley, recombination is severely suppressed in some regions (Kunzel et al. 2000). Repetitive sequences are associated with distinct chromatin modifications, and their expansion suppresses the recombination rate (Henderson 2012). Hotspots account for less than 5% of the genome region. Distal CO occurrence is 25 times greater than interstitial chiasmata (Higgins et al. 2012).

## Hotspots DNA motifs

Recombination events are correlated with the presence of specific DNA sequences (Zelkowski et al. 2019). DNA and chromatin features are associated with DSB hotspots. The popular motifs with the hotspots include CCN repeat motif, poly-A motif, and min-inverted-repeat transposable elements.

In *Arabidopsis*, DSB hotspots are correlated to CO hotspots. Three DNA motifs (A‐rich, CCN and CTT) have been found to be enriched in CO regions (Shilo et al. 2015). In maize, an associated 20‐bp‐long, GC‐rich sequence motif is similar to the CCN motif identified in *Arabidopsis* (Shilo et al. 2015). The recombination hotspot in maize is located in the bronze and *Stc1* locus (Fu et al. 2001; He and Dooner 2009) and the recombination event is significantly enriched in GC-rich regions, which is similar to the CCN motif identified in *Arabidopsis* and yeast (Gerton et al. 2000; Liu et al. 2009; Sidhu et al. 2015). In cucumber, numerous hotpot motifs are identified for DSB, including the TATA repeat (Wang et al. 2023).

## The regulation of recombination

The precise control of the frequency and distribution of meiotic recombination events remains challenging. Previous studies indicated that homologous chromosome pairing typically results in generated at least one CO event per chromosome (Bishop and Zickler 2004; Hillers 2004; Kleckner et al. 2004; Martini et al. 2006; Shinohara et al. 2008). However, a recent report showed that the absence of COs in some chromosomes in a few *F*_2_ lines in cucumber (Wang et al. 2023). Meiotic recombination distribution is uneven along chromosomes, and its regulation can be classified into chromosome-level regulation, genome-level regulation and other mechanisms (Kauppi et al. 2004; Lichten and Goldman 1995; Petes 2001; Sidhu et al. 2015).

## Chromosome level regulation

The frequency of COs increases from centromeres to telomeres, with notably low frequency observed in the telomeric region (Chen et al. 2008; Higgins et al. 2012; Liu et al. 2009; Saintenac et al. 2009, 2011; Salome et al. 2012; Sidhu et al. 2015). In most organisms, each chromosome pair usually every undergoes one or two COs (Martini et al. 2006). When more than two COs present on the homologous recombination, one CO tends to suppress the occurrence of others in nearby regions, a phenomenon known as CO interference (Hillers 2004; Jones 1984; Kleckner et al. 2004; Muller 1916). This phenomenon also appears during the DSB period. For example, the occurrence of a DSB on one chromosome of *S. cerevisiae* suppresses the frequency of DSB generation on its homolog at the same and nearby positions (Fukuda et al. 2008; Xu and Kleckner 1995).

In cucumber, however, Wang et al. (2023) observed the absence of COs on chromosomes 3,4 and 5 in the individual line Y-154, no CO on chromosome 3 and 4 in the line X-69, no CO on chromosome 7 in line Y-231, and no CO on 1,5,6 in Y-284 (Wang et al. 2023), suggesting that this regulation system may not work in cucumber.

## Genome level regulation

Recombination events tend to cluster in short specific genome regions of the genome (Marand et al. 2017). Studies in the mammalian species revealed the correlation between hotspot location and certain sequence motifs (Buard and de Massy 2007; Myers et al. 2005; Parvanov et al. 2010; Shifman et al. 2006; Smagulova et al. 2011). In humans and mice, the meiotic recombination hotspots are closely linked to the specific recognition DNA sequence of PRDM9 zinc finger protein (Borde and de Massy 2013; De Massy 2013). However, PRDM subfamilies are absent in plants (Zhang and Ma 2012). In plants, meiotic recombination hotspots tend to occur in regions close to gene promoters and terminators associated with active chromatin modifications (Choi et al. 2013; Drouaud et al. 2013; Fu et al. 2002; He et al. 2017; Wang and Copenhaver 2018). Previous studies in *Arabidopsis* indicated a consistency between recombination hotspots and DSB hotspot regions (Choi et al. 2013; Horton et al. 2012). However, recombination events in maize are only associated with the DSBs close to the genes (He and Dooner 2009; Yao et al. 2002).

## Other regulation factors

Recombination events can be influenced by extrinsic conditions, such as biotic stress, extreme temperature, chemical substances, nutrients, and UV radiation (Boyko et al. 2007; Higgins et al. 2012; Lucht et al. 2002; Mickelbart et al. 2015; Molinier et al. 2006; Phillips et al. 2015). The formation of COs relies on the ZMM protein or the endonuclease Mus81 (Berchowitz et al. 2007; Bishop and Zickler 2004; Borner et al. 2004; Santos et al. 2003; Mercier et al. 2005; Shinohara et al. 2008). Posttranslational modification could influence the activity and stability of proteins related to meiotic recombination, thereby regulating recombination events (Wang and Copenhaver 2018). DNA methylation has also implicated in regulating the formation of meiotic recombination (Buard and de Massy 2007).

The effect of temperature on miotic recombination has been reported in several species. In *Allium ursinum*, exposure to 35 °C for 30 h resulted in a detrimental effect on chromosome synapsis (Loidl 1989), whereas in barley, synapsis failed to occur at 35 °C (Higgins et al. 2012). In addition, the distribution and frequency of Chiasmata were altered when exposed to temperatures of 30 °C and 22 °C. At 30 °C, there was an increase in interstitial/proximal chiasmata, but the average number of chiasmata and COs per cell were significantly decreased (Higgins et al. 2012).

Phillips et al. (2015) found that the recombination rate during male meiosis consistently suppressed that of females. Moreover, in barley, as the temperature increased from 15 °C to 25 °C and 30 °C, the recombination rate increased during male meiosis but decreased during female meiosis. Similarly, in *Arabidopsis*, Giraut et al. (2011) demonstrated higher CO frequencies during male meiosis.

## How to increase CO frequency

### CRISPR-Cas9

The main limitation of targeted homologous recombination is DSB formation. The homologous recombination frequency can be enhanced dramatically when the DSB occurs at the target locus (Hayut et al. 2017; Puchta and Fauser 2013). Therefore, it is essential to find effective methods for inducing greater DSB formation. Sequence-specific nucleases (SSNs) are recognized for their capability to generate DSB at a specific site (Belhaj et al. 2015). The development of sequence-specific nuclease, including zinc finger nucleases (ZFNs) and transcription activator-like effector nucleases (TALENs), has already proven successful in targeted gene editing in plants (Mao et al. 2019; Podevin et al. 2013; Voytas and Gao 2014; Wang et al. 2019). However, challenges associated with the design and construction of large modular proteins have hindered their widespread adoption (Doudna and Charpentier 2014). In addition, ZFNs have shown a high fault rate during DNA sequence recognition and cleavage (Voytas 2013). In contrast, the CRISPR-Cas method has emerged as a versatile solution. In recent years, the CRISPR-Cas system, a relatively easy and powerful gene-editing tool, has achieved rapid development. Most studies have used CRISPR-Cas9 technology to edit genes in homozygous tissues, potentially increasing the occurrence of DSB. Recently, the CRISPR-Cas9 system has been used in targeted recombination in tomatoes (Hayut et al. 2017). In this study, the F_1_ hybrid seed was used for targeted DNA editing, resulting in homologous CO events. Applying the allele specific recombination analysis suggests that the homologous recombination rate can be increased by generating DSB (Hayut et al. 2017).

### Mutation

Mutating genes involved in COs is a powerful tool to increase CO frequency. *FANCM* and *RECQ4* are key players in the CO pathway, and the impact of mutations in these genes on CO rate has been extensively studied in *Arabidopsis* (Crismani et al. 2012; Seguela-Arnaud et al. 2015). For instance, *fancm* (Crismani et al. 2012) and *recq4a/b* (Seguela-Arnaud et al. 2015) mutants exhibit a nearly 3–5.9 folds increase in recombination rate (Fernandes et al. 2018b).

The AAA‐ATPase FIDGETIN‐like 1 (FIGL1) negatively regulates CO formation at the early stages (Girard et al. 2015). The *figl1* mutation enhances the CO rate by 1.5 times (Girard et al. 2015) and when combined with a*recq4a/b* double mutation, the CO rate is significantly increased (7.8‐fold) (Fernandes et al. 2018b). Remarkably, this mutant exhibits 60.7 COs per meiosis, compared to only 7.8 COs in the wild type (Fernandes et al. 2018b).

### Other approaches

Higher temperatures increase recombination rates in male meiosis in barley (Phillips et al. 2015) with CO events increasing by 40% when the temperature rose from 15 °C to 30 °C (Phillips et al. 2015). This approach can be tried in other crops to increase the recombination rate. Furthermore, DNA methylation occurs across the plant genome, regulating gene expression (Jeddeloh et al. 1998) and silencing transposable element activity (Slotkin and Martienssen 2007). Changing DNA methylation patterns has been shown to alter CO distribution in mutant plants. For instance, loss of CG methylation in *Arabidopsis* leads to changes in CO distribution (Melamed-Bessudo and Levy 2012; Mirouze et al. 2012). These studies suggest that modifying methylation patterns can remodel CO distribution in plants.

## Conclusion

While there are slight variations in CO distribution patterns, most recombination events occur toward the ends of chromosomes. In plant breeding programs, changing temperatures, creating mutations, reducing methylation patterns, and targeting CRISPR-Cas9 system can be used to regulate the recombination frequency within coldspots. With the development of whole genome sequencing, the identification of recombination spots becomes more precise. High-density genetic maps and resequencing data can be used to identify the precise location of the CO events and their associated motifs. A better understanding of CO and targeting hotspots will facilitate CO regulation in crop breeding programs.

## Data Availability

No datasets were generated or analysed during the current study.

## References

[CR1] Aklilu BB, Soderquist RS, Culligan KM (2014). Genetic analysis of the replication protein A large subunit family in *Arabidopsis* reveals unique and overlapping roles in DNA repair, meiosis and DNA replication. Nucleic Acids Res.

[CR2] Allers T, Lichten M (2001). Differential timing and control of noncrossover and crossover recombination during meiosis. Cell.

[CR3] An XJ, Deng ZY, Wang T (2011). OsSpo11-4, a rice homologue of the archaeal TopVIA protein, mediates double-strand DNA cleavage and interacts with OsTopVIB. PLoS ONE.

[CR4] Anderson LK, Doyle GG, Brigham B, Carter J, Hooker KD, Lai A, Rice M, Stack SM (2003). High-resolution crossover maps for each bivalent of *Zea mays* using recombination nodules. Genetics.

[CR5] Armstrong SJ, Jones GH (2003). Meiotic cytology and chromosome behaviour in wild-type *Arabidopsis thaliana*. J Exp Bot.

[CR6] Aylon Y, Kupiec M (2004). DSB repair: the yeast paradigm. DNA Repair.

[CR7] Azumi Y, Liu DH, Zhao DZ, Li WX, Wang GF, Hu Y, Ma H (2002). Homolog interaction during meiotic prophase I in *Arabidopsis* requires the *SOLO DANCERS* gene encoding a novel cyclin-like protein. EMBO J.

[CR8] Baker BS, Boyd JB, Carpenter ATC, Green MM, Nguyen TD, Ripoll P, Smith PD (1976). Genetic controls of meiotic recombination and somatic DNA metabolism in *Drosophila melanogaster*. P Natl Acad Sci USA.

[CR9] Barakate A, Higgins JD, Vivera S, Stephens J, Perry RM, Ramsay L, Colas I, Oakey H, Waugh R, Franklin FCH, Armstrong SJ, Halpin C (2014). The synaptonemal complex protein ZYP1 is required for imposition of meiotic crossovers in barley. Plant Cell.

[CR10] Barakate A, Arrieta M, Macaulay M, Vivera S, Davidson D, Stephens J, Orr J, Schreiber M, Ramsay L, Halpin C, Waugh R (2021). Downregulation of barley regulator of telomere elongation helicase 1 alters the distribution of meiotic crossovers. Front Plant Sci.

[CR11] Belhaj K, Chaparro-Garcia A, Kamoun S, Patron NJ, Nekrasov V (2015). Editing plant genomes with CRISPR/Cas9. Curr Opin Biotechnol.

[CR12] Berchowitz LE, Francis KE, Bey AL, Copenhaver GP (2007). The role of AtMUS81 in interference-insensitive crossovers in *A. thaliana*. Plos Genet.

[CR13] Bergerat A, deMassy B, Gadelle D, Varoutas PC, Nicolas A, Forterre P (1997). An atypical topoisomerase II from archaea with implications for meiotic recombination. Nature.

[CR14] Bishop DK (2012). Rad51, the lead in mitotic recombinational DNA repair, plays a supporting role in budding yeast meiosis. Cell Cycle.

[CR15] Bishop DK, Zickler D (2004). Early decision: meiotic crossover interference prior to stable strand exchange and synapsis. Cell.

[CR16] Bishop DK, Park D, Xu LZ, Kleckner N (1992). DMC1: a meiosis-specific yeast homolog of *E. coli* recA required for recombination, synaptonemal complex formation, and cell cycle progression. Cell.

[CR17] Blair MW, Cortes AJ, Farmer AD, Huang W, Ambachew D, Penmetsa RV, Carrasquilla-Garcia N, Assefa T, Cannon SB (2018). Uneven recombination rate and linkage disequilibrium across a reference SNP map for common bean (*Phaseolus vulgaris *L.). PLoS ONE.

[CR18] Blary A, Gonzalo A, Eber F, Berard A, Berges H, Bessoltane N, Charif D, Charpentier C, Cromer L, Fourment J, Genevriez C, Le Paslier MC, Lode M, Lucas MO, Nesi N, Lloyd A, Chevre AM, Jenczewski E (2020) FANCM limits meiotic crossovers in Brassica Crops. Front Plant Sci 11:337590

[CR19] Bleuyard JY, White CI (2004). The *Arabidopsis* homologue of Xrcc3 plays an essential role in meiosis. Embo J.

[CR20] Bleuyard JY, Gallego ME, White CI (2004). Meiotic defects in the *Arabidopsis* rad50 mutant point to conservation of the MRX complex function in early stages of meiotic recombination. Chromosoma.

[CR21] Borde V, de Massy B (2013). Programmed induction of DNA double strand breaks during meiosis: setting up communication between DNA and the chromosome structure. Curr Opin Genet Dev.

[CR22] Borde V, Lin W, Novikov E, Petrini JH, Lichten M, Nicolas A (2004). Association of Mre11p with double-strand break sites during yeast meiosis. Mol Cell.

[CR23] Borner GV, Kleckner N, Hunter N (2004). Crossover/noncrossover differentiation, synaptonemal complex formation, and regulatory surveillance at the leptotene/zygotene transition of meiosis. Cell.

[CR24] Boyko A, Kathiria P, Zemp FJ, Yao YL, Pogribny I, Kovalchuk I (2007). Transgenerational changes in the genome stability and methylation in pathogen-infected plants (Virus-induced plant genome instability). Nucleic Acids Res.

[CR25] Brown MS, Bishop DK (2015). DNA strand exchange and RecA homologs in meiosis. Csh Perspect Biol.

[CR26] Buard J, de Massy B (2007). Playing hide and seek with mammalian meiotic crossover hotspots. Trends Genet.

[CR27] Byun MY, Kim WT (2014). Suppression of OsRAD51D results in defects in reproductive development in rice (*Oryza sativa* L.). Plant J.

[CR28] Camerini-Otero RD, Hsieh P (1995). Homologous recombination proteins in prokaryotes and eukaryotes. Annu Rev Genet.

[CR29] Cannavo E, Cejka P (2014). Sae2 promotes dsDNA endonuclease activity within Mre11-Rad50-Xrs2 to resect DNA breaks. Nature.

[CR30] Chang L, Ma H, Xue HW (2009). Functional conservation of the meiotic genes SDS and RCK in male meiosis in the monocot rice. Cell Res.

[CR31] Chang YX, Gong L, Yuan WY, Li XW, Chen GX, Li XH, Zhang QF, Wu CY (2009). Replication protein A (RPA1a) is required for meiotic and somatic DNA repair but is dispensable for DNA replication and homologous recombination in rice. Plant Physiol.

[CR32] Chelysheva L, Vezon D, Chambon A, Gendrot G, Pereira L, Lemhemdi A, Vrielynck N, Le Guin S, Novatchkova M, Grelon M (2012). The *Arabidopsis* HEI10 Is a new ZMM protein related to Zip3. Plos Genet.

[CR33] Chen CB, Zhang W, Timofejeva L, Gerardin Y, Ma H (2005). The *Arabidopsis* ROCK-N-ROLLERS gene encodes a homolog of the yeast ATP-dependent DNA helicase MER3 and is required for normal meiotic crossover formation. Plant J.

[CR34] Chen SY, Tsubouchi T, Rockmill B, Sandler JS, Richards DR, Vader G, Hochwagen A, Roeder GS, Fung JC (2008). Global analysis of the meiotic crossover landscape. Dev Cell.

[CR35] Choi KH, Zhao XH, Kelly KA, Venn O, Higgins JD, Yelina NE, Hardcastle TJ, Ziolkowski PA, Copenhaver GP, Franklin FCH, McVean G, Henderson IR (2013). *Arabidopsis* meiotic crossover hot spots overlap with H2A. Z nucleosomes at gene promoters. Nat Genet.

[CR36] Cole F, Keeney S, Jasin M (2010). Evolutionary conservation of meiotic DSB proteins: more than just Spo11. Gene Dev.

[CR37] Cole F, Kauppi L, Lange J, Roig I, Wang R, Keeney S, Jasin M (2012). Homeostatic control of recombination is implemented progressively in mouse meiosis. Nat Cell Biol.

[CR38] Corredor E, Lukaszewski AJ, Pachon P, Allen DC, Naranjo T (2007). Terminal regions of wheat chromosomes select their pairing partners in meiosis. Genetics.

[CR39] Couteau F, Belzile F, Horlow C, Grandjean O, Vezon D, Doutriaux MP (1999). Random chromosome segregation without meiotic arrest in both male and female meiocytes of a dmc1 mutant of *Arabidopsis*. Plant Cell.

[CR40] Covo S, Ma WJ, Westmoreland JW, Gordenin DA, Resnick MA (2012). Understanding the origins of UV-induced recombination through manipulation of sister chromatid cohesion. Cell Cycle.

[CR41] Crismani W, Girard C, Froger N, Pradillo M, Santos JL, Chelysheva L, Copenhaver GP, Horlow C, Mercier R (2012). FANCM limits meiotic crossovers. Science.

[CR42] Da Ines O, Abe K, Goubely C, Gallego ME, White CI (2012). Differing requirements for RAD51 and DMC1 in meiotic pairing of centromeres and chromosome arms in *Arabidopsis thaliana*. Plos Genet.

[CR43] Darrier B, Rimbert H, Balfourier F, Pingault L, Josselin AA, Servin B, Navarro J, Choulet F, Paux E, Sourdille P (2017). High-resolution mapping of crossover events in the hexaploid wheat genome suggests a universal recombination mechanism. Genetics.

[CR44] De Massy B (2013). Initiation of meiotic recombination: how and where? Conservation and specificities among eukaryotes. Annu Rev Genet.

[CR45] De Maagd RA, Loonen A, Chouaref J, Pele A, Meijer-Dekens F, Fransz P, Bai YL (2020). CRISPR/Cas inactivation of RECQ4 increases homeologous crossovers in an interspecific tomato hybrid. Plant Biotechnol J.

[CR46] De Muyt A, Vezon D, Gendrot G, Gallois JL, Stevens R, Grelon M (2007). AtPRD1 is required for meiotic double strand break formation in *Arabidopsis thaliana*. Embo J.

[CR47] De Muyt A, Pereira L, Vezon D, Chelysheva L, Gendrot G, Chambon A, Laine-Choinard S, Pelletier G, Mercier R, Nogue F, Grelon M (2009). A high throughput genetic screen identifies new early meiotic recombination functions in *Arabidopsis thaliana*. Plos Genet.

[CR48] Deng ZY, Wang T (2007). *OsDMC1* is required for homologous pairing in *Oryza sativa*. Plant Mol Biol.

[CR49] Diaz RL, Alcid AD, Berger JM, Keeney S (2002). Identification of residues in yeast Spo11p critical for meiotic DNA double-strand break formation. Mol Cell Biol.

[CR50] Dion E, Li LL, Jean M, Beizile F (2007). An *Arabidopsis* MLH1 mutant exhibits reproductive defects and reveals a dual role for this gene in mitotic recombination. Plant J.

[CR51] Doudna JA, Charpentier E (2014). The new frontier of genome engineering with CRISPR-Cas9. Science.

[CR52] Drouaud J, Khademian H, Giraut L, Zanni V, Bellalou S, Henderson IR, Falque M, Mezard C (2013). Contrasted patterns of crossover and non-crossover at *Arabidopsis thaliana* meiotic recombination hotspots. Plos Genet.

[CR53] Duroc Y, Lemhemdi A, Larcheveque C, Hurel A, Cuacos M, Cromer L, Horlow C, Armstrong SJ, Chelysheva L, Mercier R (2014). The kinesin AtPSS1 promotes synapsis and is required for proper crossover distribution in meiosis. Plos Genet.

[CR54] Erayman M, Sandhu D, Sidhu D, Dilbirligi M, Baenziger PS, Gill KS (2004). Demarcating the gene-rich regions of the wheat genome. Nucleic Acids Res.

[CR55] Fedoroff N (2000). Transposons and genome evolution in plants. P Natl Acad Sci USA.

[CR56] Fernandes JB, Duhamel M, Seguela-Arnaud M, Froger N, Girard C, Choinard S, Solier V, De Winne N, De Jaeger G, Gevaert K, Andrey P, Grelon M, Guerois R, Kumar R, Mercier R (2018). FIGL1 and its novel partner FLIP form a conserved complex that regulates homologous recombination. Plos Genet.

[CR57] Fernandes JB, Seguela-Arnaud M, Larcheveque C, Lloyd AH, Mercier R (2018). Unleashing meiotic crossovers in hybrid plants. P Natl Acad Sci USA.

[CR58] Fraune J, Schramm S, Alsheimer M, Benavente R (2012). The mammalian synaptonemal complex: protein components, assembly and role in meiotic recombination. Exp Cell Res.

[CR59] Fu HH, Park WK, Yan XH, Zheng ZW, Shen BZ, Dooner HK (2001). The highly recombinogenic *bz* locus lies in an unusually gene-rich region of the maize genome. P Natl Acad Sci USA.

[CR60] Fu HH, Zheng ZW, Dooner HK (2002). Recombination rates between adjacent genic and retrotransposon regions in maize vary by 2 orders of magnitude. P Natl Acad Sci USA.

[CR61] Fu M, Wang C, Xue FY, Higgins J, Chen MJ, Zhang DB, Liang WQ (2016). The DNA topoisomerase VI-B subunit *OsMTOPVIB* is essential for meiotic recombination initiation in rice. Mol Plant.

[CR62] Fukuda T, Kugou K, Sasanuma H, Shibata T, Ohta K (2008). Targeted induction of meiotic double-strand breaks reveals chromosomal domain-dependent regulation of Spo11 and interactions among potential sites of meiotic recombination. Nucleic Acids Res.

[CR63] Gadelle D, Filee J, Buhler C, Forterre P (2003). Phylogenomics of type II DNA topoisomerases. BioEssays.

[CR64] Garcia V, Phelps SEL, Gray S, Neale MJ (2011). Bidirectional resection of DNA double-strand breaks by Mre11 and Exo1. Nature.

[CR65] Gardiner LJ, Wingen LU, Bailey P, Joynson R, Brabbs T, Wright J, Higgins JD, Hall N, Griffiths S, Clavijo BJ, Hall A (2019). Analysis of the recombination landscape of hexaploid bread wheat reveals genes controlling recombination and gene conversion frequency. Genome Biol.

[CR66] Gerton JL, DeRisi J, Shroff R, Lichten M, Brown PO, Petes TD (2000). Global mapping of meiotic recombination hotspots and coldspots in the yeast *Saccharomyces cerevisiae*. P Natl Acad Sci USA.

[CR67] Gilbert SF, Barresi MJF (2016). Developmental biology.

[CR68] Girard C, Chelysheva L, Choinard S, Froger N, Macaisne N, Lehmemdi A, Mazel J, Crismani W, Mercier R (2015). AAA-ATPase FIDGETIN-LIKE 1 and helicase FANCM antagonize meiotic crossovers by distinct mechanisms. Plos Genet.

[CR69] Giraut L, Falque M, Drouaud J, Pereira L, Martin OC, Mezard C (2011). Genome-wide crossover distribution in *Arabidopsis thaliana* meiosis reveals sex-specific patterns along chromosomes. Plos Genet.

[CR70] Gore MA, Chia JM, Elshire RJ, Sun Q, Ersoz ES, Hurwitz BL, Peiffer JA, McMullen MD, Grills GS, Ross-Ibarra J, Ware DH, Buckler ES (2009). A first-generation haplotype map of maize. Science.

[CR71] Gratia JP (2017). Genetic recombinational events in prokaryotes and their viruses: insight into the study of evolution and biodiversity. Anton Leeuw Int J G.

[CR72] Grelon M, Vezon D, Gendrot G, Pelletier G (2001). AtSPO11-1 is necessary for efficient meiotic recombination in plants. EMBO J.

[CR73] Griffiths S, Sharp R, Foote TN, Bertin I, Wanous M, Reader S, Colas I, Moore G (2006). Molecular characterization of *Ph1* as a major chromosome pairing locus in polyploid wheat. Nature.

[CR74] Grindley NDF, Whiteson KL, Rice PA (2006). Mechanisms of site-specific recombination. Annu Rev Biochem.

[CR75] Hartl DL, Ruvolo M (2012). Genetics : analysis of genes and genomes.

[CR76] Hartung F, Puchta H (2000). Molecular characterisation of two paralogous SPO11 homologues in *Arabidopsis thaliana*. Nucleic Acids Res.

[CR77] Hartung F, Suer S, Bergmann T, Puchta H (2006). The role of *AtMUS81* in DNA repair and its genetic interaction with the helicase *AtRecQ4A*. Nucleic Acids Res.

[CR78] Hartung F, Wurz-Wildersinn R, Fuchs J, Schubert I, Suer S, Puchta H (2007). The catalytically active tyrosine residues of both SPO11-1 and SPO11-2 are required for meiotic double-strand break induction in *Arabidopsis*. Plant Cell.

[CR79] Hayashi M, Mlynarczyk-Evans S, Villeneuve AM (2010). The synaptonemal complex shapes the crossover landscape through cooperative assembly, crossover promotion and crossover inhibition during *Caenorhabditis elegans* meiosis. Genetics.

[CR80] Hayut SF, Bessudo CM, Levy AA (2017). Targeted recombination between homologous chromosomes for precise breeding in tomato. Nat Commun.

[CR81] He LM, Dooner HK (2009). Haplotype structure strongly affects recombination in a maize genetic interval polymorphic for *Helitron* and retrotransposon insertions. P Natl Acad Sci USA.

[CR82] He Y, Wang MH, Dukowic-Schulze S, Zhou A, Tiang CL, Shilo S, Sidhu GK, Eichten S, Bradbury P, Springer NM, Buckler ES, Levy AA, Sun Q, Pillardy J, Kianian PMA, Kianian SF, Chen CB, Pawlowski WP (2017). Genomic features shaping the landscape of meiotic double-strand-break hotspots in maize. P Natl Acad Sci USA.

[CR83] Henderson IR (2012). Control of meiotic recombination frequency in plant genomes. Curr Opin Plant Biol.

[CR84] Hernandez-Hernandez A, Masich S, Fukuda T, Kouznetsova A, Sandin S, Daneholt B, Hoog C (2016). The central element of the synaptonemal complex in mice is organized as a bilayered junction structure. J Cell Sci.

[CR85] Heyting C (1996). Synaptonemal complexes: structure and function. Curr Opin Cell Biol.

[CR86] Higgins JD, Armstrong SJ, Franklin FCH, Jones GH (2004). The *Arabidopsis MutS* homolog *AtMSH4* functions at an early step in recombination: evidence for two classes of recombination in *Arabidopsis*. Gene Dev.

[CR87] Higgins JD, Sanchez-Moran E, Armstrong SJ, Jones GH, Franklin FCH (2005). The *Arabidopsis* synaptonemal complex protein ZYP1 is required for chromosome synapsis and normal fidelity of crossing over. Gene Dev.

[CR88] Higgins JD, Buckling EF, Franklin FCH, Jones GH (2008). Expression and functional analysis of *AtMUS81* in *Arabidopsis* meiosis reveals a role in the second pathway of crossing-over. Plant J.

[CR89] Higgins JD, Vignard J, Mercier R, Pugh AG, Franklin FCH, Jones GH (2008). AtMSH5 partners AtMSH4 in the class I meiotic crossover pathway in *Arabidopsis thaliana*, but is not required for synapsis. Plant J.

[CR90] Higgins JD, Perry RM, Barakate A, Ramsay L, Waugh R, Halpin C, Armstrong SJ, Franklin FCH (2012). Spatiotemporal asymmetry of the meiotic program underlies the predominantly distal distribution of meiotic crossovers in barley. Plant Cell.

[CR91] Higgins JD, Osman K, Jones GH, Franklin FC (2014). Factors underlying restricted crossover localization in barley meiosis. Annu Rev Genet.

[CR92] Hillers KJ (2004). Crossover interference. Curr Biol.

[CR93] Hillers KJ, Jantsch V, Martinez-Perez E, Yanowitz JL (2017). Meiosis. Wormbook.

[CR94] Horton MW, Hancock AM, Huang YS, Toomajian C, Atwell S, Auton A, Muliyati NW, Platt A, Sperone FG, Vilhjalmsson BJ, Nordborg M, Borevitz JO, Bergelson J (2012). Genome-wide patterns of genetic variation in worldwide *Arabidopsis thaliana* accessions from the RegMap panel. Nat Genet.

[CR95] Hunter N (2015). Meiotic recombination: the essence of heredity. Cold Spring Harb Perspect Biol.

[CR96] Hunter N, Kleckner N (2001). The single-end invasion: an asymmetric intermediate at the double-strand break to double-holliday junction transition of meiotic recombination. Cell.

[CR97] Jackson N, Sanchez-Moran E, Buckling E, Armstrong SJ, Jones GH, Franklin FCH (2006). Reduced meiotic crossovers and delayed prophase I progression in AtMLH3-deficient *Arabidopsis*. Embo J.

[CR98] Jayakodi M, Padmarasu S, Haberer G, Bonthala VS, Gundlach H, Monat C, Lux T, Kamal N, Lang DI, Himmelbach A, Ens J, Zhang XQ, Angessa TT, Zhou GF, Tan C, Hill C, Wang PH, Schreiber M, Boston LB, Plott C, Jenkins J, Guo Y, Fiebig A, Budak H, Xu DD, Zhang J, Wang CC, Grimwood J, Schmutz J, Guo GG, Zhang GP, Mochida K, Hirayama T, Sato K, Chalmers KJ, Langridge P, Waugh R, Pozniak CJ, Scholz U, Mayer KFX, Spannagl M, Li CD, Mascher M, Stein N (2020). The barley pan-genome reveals the hidden legacy of mutation breeding. Nature.

[CR99] Jeddeloh JA, Bender J, Richards EJ (1998). The DNA methylation locus *DDM1* is required for maintenance of gene silencing in *Arabidopsis*. Gene Dev.

[CR100] Ji JH, Tang D, Wang KJ, Wang M, Che LX, Li M, Cheng ZK (2012). The role of OsCOM1 in homologous chromosome synapsis and recombination in rice meiosis. Plant J.

[CR101] Ji JH, Tang D, Wang M, Li YF, Zhang L, Wang KJ, Li M, Cheng ZK (2013). MRE11 is required for homologous synapsis and DSB processing in rice meiosis. Chromosoma.

[CR102] Jolivet S, Vezon D, Froger N, Mercier R (2006). Non conservation of the meiotic function of the Ski8/Rec103 homolog in *Arabidopsis*. Genes Cells.

[CR103] Jones GH (1984). The control of chiasma distribution. Symp Soc Exp Biol.

[CR104] Jones GH, Franklin FCH (2006). Meiotic crossing-over: obligation and interference. Cell.

[CR105] Kauppi L, Jeffreys AJ, Keeney S (2004). Where the crossovers are: recombination distributions in mammals. Nat Rev Genet.

[CR106] Keeney S (2008). Spo11 and the formation of DNA double-strand breaks in meiosis. Genome Dyn Stab.

[CR107] Keeney S, Neale MJ (2006). Initiation of meiotic recombination by formation of DNA double-strand breaks: mechanism and regulation. Biochem Soc T.

[CR108] Keeney S, Giroux CN, Kleckner N (1997). Meiosis-specific DNA double-strand breaks are catalyzed by Spo11, a member of a widely conserved protein family. Cell.

[CR109] Kianian PMA, Wang MH, Simons K, Ghavami F, He Y, Dukowic-Schulze S, Sundararajan A, Sun Q, Pillardy J, Mudge J, Chen CB, Kianian SF, Pawlowski WP (2018). High-resolution crossover mapping reveals similarities and differences of male and female recombination in maize. Nat Commun.

[CR110] Kleckner N (1996). Meiosis: how could it work?. P Natl Acad Sci USA.

[CR111] Kleckner N, Zickler D, Jones GH, Dekker J, Padmore R, Henle J, Hutchinson J (2004). A mechanical basis for chromosome function. P Natl Acad Sci USA.

[CR112] Knoll A, Higgins JD, Seeliger K, Reha SJ, Dangel NJ, Bauknecht M, Schropfer S, Franklin FCH, Puchta H (2012). The Fanconi anemia ortholog FANCM ensures ordered homologous recombination in both somatic and meiotic cells in *Arabidopsis*. Plant Cell.

[CR113] Kou YJ, Chang YX, Li XH, Xiao JH, Wang SP (2012). The rice *RAD51C* gene is required for the meiosis of both female and male gametocytes and the DNA repair of somatic cells. J Exp Bot.

[CR114] Kouznetsova A, Benavente R, Pastink A, Hoog C (2011). Meiosis in mice without a synaptonemal complex. PLoS ONE.

[CR115] Kunzel G, Korzun L, Meister A (2000). Cytologically integrated physical restriction fragment length polymorphism maps for the barley genome based on translocation breakpoints. Genetics.

[CR116] Kuromori T, Azumi Y, Hayakawa S, Kamiya A, Imura Y, Wada T, Shinozaki K (2008). Homologous chromosome pairing is completed in crossover defective atzip4 mutant. Biochem Bioph Res Co.

[CR117] Lake CM, Hawley RS (2016). Becoming a crossover-competent DSB. Semin Cell Dev Biol.

[CR118] Lam I, Keeney S (2015). Mechanism and regulation of meiotic recombination initiation. Csh Perspect Biol.

[CR119] Lefrancois P, Rockmill B, Xie P, Roeder GS, Snyder M (2016). Multiple pairwise analysis of non-homologous centromere coupling reveals preferential chromosome size-dependent interactions and a role for bouquet formation in establishing the interaction pattern. Plos Genet.

[CR120] Lenormand T, Dutheil J (2005). Recombination difference between sexes: a role for haploid selection. Plos Biol.

[CR121] Li J, Hooker GW, Roeder GS (2006). *Saccharomyces cerevisiae* Mer2, Mei4 and Rec114 form a complex required for meiotic double-strand break formation. Genetics.

[CR122] Li J, Harper LC, Golubovskaya I, Wang CR, Weber D, Meeley RB, McElver J, Bowen B, Cande WZ, Schnable PS (2007). Functional analysis of maize RAD51 in meiosis and double-strand break repair. Genetics.

[CR123] Li XW, Chang YX, Xin XD, Zhu CM, Li XH, Higgins JD, Wu CY (2013). Replication protein A2c coupled with replication protein A1c regulates crossover formation during meiosis in rice. Plant Cell.

[CR124] Li X, Li L, Yan JB (2015). Dissecting meiotic recombination based on tetrad analysis by single-microspore sequencing in maize. Nat Commun.

[CR125] Lian QC, Solier V, Walkemeier B, Durand S, Huettel B, Schneeberger K, Mercier R (2022). The megabase-scale crossover landscape is largely independent of sequence divergence. Nat Commun.

[CR126] Lichten M (2001). Meiotic recombination: breaking the genome to save it. Curr Biol.

[CR127] Lichten M, Goldman ASH (1995). Meiotic recombination hotspots. Annu Rev Genet.

[CR128] Liu SZ, Yeh CT, Ji TM, Ying K, Wu HY, Tang HM, Fu Y, Nettleton D, Schnable PS (2009). Mu transposon insertion sites and meiotic recombination events co-localize with epigenetic marks for open chromatin across the maize genome. PLoS Genet.

[CR129] Liu Y, Deng YT, Li G, Zhao J (2013). Replication factor C1 (RFC1) is required for double-strand break repair during meiotic homologous recombination in *Arabidopsis*. Plant J.

[CR130] Loidl J (1989). Effects of elevated-temperature on meiotic chromosome synapsis in *Allium ursinum*. Chromosoma.

[CR131] Lu PL, Han XW, Qi J, Yang JG, Wijeratne AJ, Li T, Ma H (2012). Analysis of *Arabidopsis* genome-wide variations before and after meiosis and meiotic recombination by resequencing Landsberg *erecta* and all four products of a single meiosis. Genome Res.

[CR132] Lucht JM, Mauch-Mani B, Steiner HY, Metraux JP, Ryals J, Hohn B (2002). Pathogen stress increases somatic recombination frequency in *Arabidopsis*. Nat Genet.

[CR133] Lukaszewski AJ (1992). A comparison of physical distribution of recombination in chromosome 1R in diploid rye and in hexaploid triticale. Theor Appl Genet.

[CR134] Luo Q, Tang D, Wang M, Luo WX, Zhang L, Qin BX, Shen Y, Wang KJ, Li YF, Cheng ZK (2013). The role of OsMSH5 in crossover formation during rice meiosis. Mol Plant.

[CR135] Ma X, Fan L, Zhang ZF, Yang X, Liu YC, Ma YM, Pan Y, Zhou GA, Zhang M, Ning HL, Kong FJ, Ma JK, Liu SL, Tian ZX (2023). Global dissection of the recombination landscape in soybean using a high-density 600K SoySNP array. Plant Biotechnol J.

[CR136] Maleki S, Neale MJ, Arora C, Henderson KA, Keeney S (2007). Interactions between Mei4, Rec114, and other proteins required for meiotic DNA double-strand break formation in *Saccharomyces cerevisiae*. Chromosoma.

[CR137] Mancera E, Bourgon R, Brozzi A, Huber W, Steinmetz LM (2008). High-resolution mapping of meiotic crossovers and non-crossovers in yeast. Nature.

[CR138] Mao YF, Botella JR, Liu YG, Zhu JK (2019). Gene editing in plants: progress and challenges. Natl Sci Rev.

[CR139] Marand AP, Jansky SH, Zhao HN, Leisner CP, Zhu XB, Zeng ZX, Crisovan E, Newton L, Hamernik AJ, Veilleux RE, Buell CR, Jiang JM (2017). Meiotic crossovers are associated with open chromatin and enriched with *Stowaway* transposons in potato. Genome Biol.

[CR140] Martinez-Perez E, Colaiacovo MP (2009). Distribution of meiotic recombination events: talking to your neighbors. Curr Opin Genet Dev.

[CR141] Martini E, Diaz RL, Hunter N, Keeney S (2006). Crossover homeostasis in yeast meiosis. Cell.

[CR142] McConaughy S, Amundsen K, Song QJ, Pantalone V, Hyten D (2023). Recombination hotspots in soybean [*Glycine max* (L.) Merr.]. G3 Genes Genomes Genet.

[CR143] McMahill MS, Sham CW, Bishop DK (2007). Synthesis-dependent strand annealing in meiosis. PLoS Biol.

[CR144] Melamed-Bessudo C, Levy AA (2012). Deficiency in DNA methylation increases meiotic crossover rates in euchromatic but not in heterochromatic regions in *Arabidopsis*. P Natl Acad Sci USA.

[CR145] Mercier R, Jolivet S, Vezon D, Huppe E, Chelysheva L, Giovanni M, Nogue F, Doutriaux MP, Horlow C, Grelon M, Mezard C (2005). Two meiotic crossover classes cohabit in *Arabidopsis*: one is dependent on MER3, whereas the other one is not. Curr Biol.

[CR146] Mercier R, Mezard C, Jenczewski E, Macaisne N, Grelon M (2015). The molecular biology of meiosis in plants. Annu Rev Plant Biol.

[CR147] Miao CB, Tang D, Zhang HG, Wang M, Li YF, Tang SZ, Yu HX, Gu MH, Cheng ZK (2013). Central region component1, a novel synaptonemal complex component, is essential for meiotic recombination initiation in rice. Plant Cell.

[CR148] Mickelbart MV, Hasegawa PM, Bailey-Serres J (2015). Genetic mechanisms of abiotic stress tolerance that translate to crop yield stability. Nat Rev Genet.

[CR149] Mirouze M, Lieberman-Lazarovich M, Aversano R, Bucher E, Nicolet J, Reinders J, Paszkowski J (2012). Loss of DNA methylation affects the recombination landscape in *Arabidopsis*. P Natl Acad Sci USA.

[CR150] Mitchel K, Zhang HS, Welz-Voegele C, Jinks-Robertson S (2010). Molecular structures of crossover and noncrossover intermediates during gap repair in yeast: implications for recombination. Mol Cell.

[CR151] Molinier J, Ries G, Zipfel C, Hohn B (2006). Transgeneration memory of stress in plants. Nature.

[CR152] Muller HJ (1916). The mechanism of crossing-over. Am Nat.

[CR153] Murakami H, Keeney S (2008). Regulating the formation of DNA double-strand breaks in meiosis. Gene Dev.

[CR154] Myers S, Bottolo L, Freeman C, McVean G, Donnelly P (2005). A fine-scale map of recombination rates and hotspots across the human genome. Science.

[CR155] Neale MJ, Pan J, Keeney S (2005). Endonucleolytic processing of covalent protein-linked DNA double-strand breaks. Nature.

[CR156] Nichols MD, DeAngelis K, Keck JL, Berger JM (1999). Structure and function of an archaeal topoisomerase VI subunit with homology to the meiotic recombination factor Spo11. EMBO J.

[CR157] Nimonkar AV, Sica RA, Kowalczykowski SC (2009). Rad52 promotes second-end DNA capture in double-stranded break repair to form complement-stabilized joint molecules. P Natl Acad Sci USA.

[CR158] Nonomura KL, Nakano M, Fukuda T, Eiguchi M, Miyao A, Hirochika H, Kurata N (2004). The novel gene *HOMOLOGOUS PAIRING ABERRATION IN RICE MEIOSIS1* of rice encodes a putative coiled-coil protein required for homologous chromosome pairing in meiosis. Plant Cell.

[CR159] Osman K, Higgins JD, Sanchez-Moran E, Armstrong SJ, Franklin FC (2011). Pathways to meiotic recombination in *Arabidopsis thaliana*. New Phytol.

[CR160] Page SL, Hawley RS (2004). The genetics and molecular biology of the synaptonemal complex. Annu Rev Cell Dev Biol.

[CR161] Paigen K, Petkov P (2010). Mammalian recombination hot spots: properties, control and evolution. Nat Rev Genet.

[CR162] Pan J, Sasaki M, Kniewel R, Murakami H, Blitzblau HG, Tischfield SE, Zhu X, Neale MJ, Jasin M, Socci ND, Hochwagen A, Keeney S (2011). A hierarchical combination of factors shapes the genome-wide topography of yeast meiotic recombination initiation. Cell.

[CR163] Pannunzio NR, Watanabe G, Lieber MR (2018). Nonhomologous DNA end-joining for repair of DNA double-strand breaks. J Biol Chem.

[CR164] Panoli AP, Ravi M, Sebastian J, Nishal B, Reddy TV, Marimuthu MPA, Subbiah V, Vijaybhaskar V, Siddiqi I (2006). At*MND1* is required for homologous pairing during meiosis in *Arabidopsis*. Bmc Mol Biol.

[CR165] Paques F, Haber JE (1999). Multiple pathways of recombination induced by double-strand breaks in *Saccharomyces cerevisiae*. Microbiol Mol Biol R.

[CR166] Parvanov ED, Petkov PM, Paigen K (2010). *Prdm9* controls activation of mammalian recombination hotspots. Science.

[CR167] Paul P, Nag D, Chakraborty S (2016). Recombination hotspots: models and tools for detection. DNA Repair (amst).

[CR168] Pawlowski WP, Golubovskaya IN, Cande WZ (2003). Altered nuclear distribution of recombination protein RAD51 in maize mutants suggests the involvement of RAD51 in meiotic homology recognition. Plant Cell.

[CR169] Pawlowski WP, Golubovskaya IN, Timofejeva L, Meeley RB, Sheridan WF, Cande WZ (2004). Coordination of meiotic recombination, pairing, and synapsis by PHS1. Science.

[CR170] Petes TD (2001). Meiotic recombination hot spots and cold spots. Nat Rev Genet.

[CR171] Phillips D, Nibau C, Ramsay L, Waugh R, Jenkins G (2010). Development of a molecular cytogenetic recombination assay for barley. Cytogenet Genome Res.

[CR172] Phillips D, Jenkins G, Macaulay M, Nibau C, Wnetrzak J, Fallding D, Colas I, Oakey H, Waugh R, Ramsay L (2015). The effect of temperature on the male and female recombination landscape of barley. New Phytol.

[CR173] Podevin N, Davies HV, Hartung F, Nogue F, Casacuberta JM (2013). Site-directed nucleases: a paradigm shift in predictable, knowledge-based plant breeding. Trends Biotechnol.

[CR174] Puchta H, Fauser F (2013). Gene targeting in plants: 25 years later. Int J Dev Biol.

[CR175] Puizina J, Siroky J, Mokros P, Schweizer D, Riha K (2004). Mre11 deficiency in *Arabidopsis* is associated with chromosomal instability in somatic cells and Spo11-dependent genome fragmentation during meiosis. Plant Cell.

[CR176] Ribeiro J, Abby E, Livera G, Martini E (2016). RPA homologs and ssDNA processing during meiotic recombination. Chromosoma.

[CR177] Rice WR (2002). Experimental tests of the adaptive significance of sexual recombination. Nat Rev Genet.

[CR178] Robert T, Nore A, Brun C, Maffre C, Crimi B, Bourbon HM, de Massy B (2016). The TopoVIB-Like protein family is required for meiotic DNA double-strand break formation. Science.

[CR179] Rodgers-Melnick E, Bradbury PJ, Elshire RJ, Glaubitz JC, Acharya CB, Mitchell SE, Li CH, Li YX, Buckler ES (2015). Recombination in diverse maize is stable, predictable, and associated with genetic load. P Natl Acad Sci USA.

[CR180] Ronceret A, Doutriaux MP, Golubovskaya IN, Pawlowski WP (2009). PHS1 regulates meiotic recombination and homologous chromosome pairing by controlling the transport of RAD50 to the nucleus. P Natl Acad Sci USA.

[CR181] Rosu S, Libuda DE, Villeneuve AM (2011). Robust crossover assurance and regulated interhomolog access maintain meiotic crossover number. Science.

[CR182] Saintenac C, Falque M, Martin OC, Paux E, Feuillet C, Sourdille P (2009). Detailed recombination studies along chromosome 3B provide new insights on crossover distribution in wheat (*Triticum aestivum* L.). Genetics.

[CR183] Saintenac C, Faure S, Remay A, Choulet F, Ravel C, Paux E, Balfourier F, Feuillet C, Sourdille P (2011). Variation in crossover rates across a 3-Mb contig of bread wheat (*Triticum aestivum*) reveals the presence of a meiotic recombination hotspot. Chromosoma.

[CR184] Salome PA, Bomblies K, Fitz J, Laitinen RAE, Warthmann N, Yant L, Weigel D (2012). The recombination landscape in *Arabidopsis thaliana* F-2 populations. Heredity.

[CR185] Sanchez-Moran E, Armstrong SJ, Santos JL, Franklin FCH, Jones GH (2002). Variation in chiasma frequency among eight accessions of *Arabidopsis thaliana*. Genetics.

[CR186] Sanchez-Moran E, Santos JL, Jones GH, Franklin FCH (2007). ASY1 mediates AtDMC1-dependent interhomolog recombination during meiosis in *Arabidopsis*. Gene Dev.

[CR187] Santos TDL, Hunter N, Lee C, Larkin B, Loidl J, Hollingsworth NM (2003). The Mus81/Mms4 endonuclease acts independently of double-Holliday junction resolution to promote a distinct subset of crossovers during meiosis in budding yeast. Genetics.

[CR188] Schnable PS, Hsia AP, Nikolau BJ (1998). Genetic recombination in plants. Curr Opin Plant Biol.

[CR189] Schramm S, Fraune J, Naumann R, Hernandez-Hernandez A, Hoog C, Cooke HJ, Alsheimer M, Benavente R (2011). A novel mouse synaptonemal complex protein is essential for loading of central element proteins, recombination, and fertility. PLoS Genet.

[CR190] Seguela-Arnaud M, Crismani W, Larcheveque C, Mazel J, Froger N, Choinard S, Lemhemdi A, Macaisne N, Van Leene J, Gevaert K, De Jaeger G, Chelysheva L, Mercier R (2015). Multiple mechanisms limit meiotic crossovers: TOP3 alpha and two BLM homologs antagonize crossovers in parallel to FANCM. P Natl Acad Sci USA.

[CR191] Seguela-Arnaud M, Choinard S, Larcheveque C, Girard C, Froger N, Crismani W, Mercier R (2017). RMI1 and TOP3 alpha limit meiotic CO formation through their C-terminal domains. Nucleic Acids Res.

[CR192] Shen Y, Tang D, Wang KJ, Wang M, Huang J, Luo WX, Luo Q, Hong LL, Li M, Cheng ZK (2012). ZIP4 in homologous chromosome synapsis and crossover formation in rice meiosis. J Cell Sci.

[CR193] Shen C, Li XM, Zhang RT, Lin ZX (2017). Genome-wide recombination rate variation in a recombination map of cotton. PLoS ONE.

[CR194] Shifman S, Bell JT, Copley RR, Taylor MS, Williams RW, Mott R, Flint J (2006). A high-resolution single nucleotide polymorphism genetic map of the mouse genome. PLoS Biol.

[CR195] Shilo S, Melamed-Bessudo C, Dorone Y, Barkai N, Levy AA (2015). DNA crossover motifs associated with epigenetic modifications delineate open chromatin regions in *Arabidopsis*. Plant Cell.

[CR196] Shingu Y, Mikawa T, Onuma M, Hirayama T, Shibata T (2010). A DNA-binding surface of SPO11-1, an *Arabidopsis* SPO11 orthologue required for normal meiosis. FEBS J.

[CR197] Shinohara A, Ogawa H, Ogawa T (1992). Rad51 protein involved in repair and recombination in *S. cerevisiae* is a RecA-like protein. Cell.

[CR198] Shinohara M, Oh SD, Hunter N, Shinohara A (2008). Crossover assurance and crossover interference are distinctly regulated by the ZMM proteins during yeast meiosis. Nat Genet.

[CR199] Shultz RW, Tatineni VM, Hanley-Bowdoin L, Thompson WF (2007). Genome-wide analysis of the core DNA replication machinery in the higher plants *Arabidopsis* and rice. Plant Physiol.

[CR200] Siaud N, Dray E, Gy I, Gerard E, Takvorian N, Doutriaux MP (2004). Brca2 is involved in meiosis in *Arabidopsis thaliana* as suggested by its interaction with Dmc1. Embo J.

[CR201] Sidhu GK, Fang C, Olson MA, Falque M, Martin OC, Pawlowski WP (2015). Recombination patterns in maize reveal limits to crossover homeostasis. P Natl Acad Sci USA.

[CR202] Slotkin RK, Martienssen R (2007). Transposable elements and the epigenetic regulation of the genome. Nat Rev Genet.

[CR203] Smagulova F, Gregoretti IV, Brick K, Khil P, Camerini-Otero RD, Petukhova GV (2011). Genome-wide analysis reveals novel molecular features of mouse recombination hotspots. Nature.

[CR204] Soustelle C, Vedel M, Kolodner R, Nicolas A (2002). Replication protein A is required for meiotic recombination in *Saccharomyces cerevisiae*. Genetics.

[CR205] Stacey NJ, Kuromori T, Azumi Y, Roberts G, Breuer C, Wada T, Maxwell A, Roberts K, Sugimoto-Shirasu K (2006). *Arabidopsis* SPO11-2 functions with SPO11-1 in meiotic recombination. Plant J.

[CR206] Stapley J, Feulner PGD, Johnston SE, Santure AW, Smadja CM (2017). Recombination: the good, the bad and the variable. Philos Trans R Soc B Biol Sci.

[CR207] Su H, Cheng ZH, Huang JY, Lin J, Copenhaver GP, Ma H, Wang YX (2017). *Arabidopsis* RAD51, RAD51C and XRCC3 proteins form a complex and facilitate RAD51 localization on chromosomes for meiotic recombination. PLoS Genet.

[CR208] Sugiyama T, Kantake N, Wu Y, Kowalczykowski SC (2006). Rad52-mediated DNA annealing after Rad51-mediated DNA strand exchange promotes second ssDNA capture. EMBO J.

[CR209] Szostak JW, Orrweaver TL, Rothstein RJ, Stahl FW (1983). The double-strand-break repair model for recombination. Cell.

[CR210] Taiz L, Zeiger E, Møller IM, Murphy AS (2015). Plant physiology and development.

[CR211] Tang D, Miao CB, Li YF, Wang HJ, Liu XF, Yu HX, Cheng ZK (2014). OsRAD51C is essential for double-strand break repair in rice meiosis. Front Plant Sci.

[CR212] Tang Y, Yin ZN, Zeng YJ, Zhang QX, Chen LQ, He Y, Lu PL, Ye D, Zhang XQ (2017). MTOPVIB interacts with AtPRD1 and plays important roles in formation of meiotic DNA double-strand breaks in *Arabidopsis*. Sci Rep.

[CR213] Uanschou C, Siwiec T, Pedrosa-Harand A, Kerzendorfer C, Sanchez-Moran E, Novatchkova M, Akimcheva S, Woglar A, Klein F, Schlogelhofer P (2007). A novel plant gene essential for meiosis is related to the human *CtIP* and the yeast *COM1/SAE2* gene. EMBO J.

[CR214] Uanschou C, Ronceret A, Von Harder M, De Muyt A, Vezon D, Pereira L, Chelysheva L, Kobayashi W, Kurumizaka H, Schlogelhofer P, Grelon M (2013). Sufficient amounts of functional HOP2/MND1 complex promote interhomolog DNA repair but are dispensable for intersister DNA repair during meiosis in *Arabidopsis*. Plant Cell.

[CR215] Vignard J, Siwiec T, Chelysheva L, Vrielynck N, Gonord F, Armstrong SJ, Schlogelhofer P, Mercier R (2007). The interplay of RecA-related proteins and the MND1-HOP2 complex during meiosis in *Arabidopsis thaliana*. PLoS Genet.

[CR216] Voytas DF (2013). Plant genome engineering with sequence-specific nucleases. Annu Rev Plant Biol.

[CR217] Voytas DF, Gao C (2014). Precision genome engineering and agriculture: opportunities and regulatory challenges. PLoS Biol.

[CR218] Vrielynck N, Chambon A, Vezon D, Pereira L, Chelysheva L, De Muyt A, Mezard C, Mayer C, Grelon M (2016). A DNA topoisomerase VI-like complex initiates meiotic recombination. Science.

[CR219] Wang YX, Copenhaver GP (2018). Meiotic recombination: mixing it up in plants. Annu Rev Plant Biol.

[CR220] Wang X, Haber JE (2004). Role of *Saccharomyces* single-stranded DNA-binding protein RPA in the strand invasion step of double-strand break repair. PLoS Biol.

[CR221] Wang KJ, Wang M, Tang D, Shen Y, Miao CB, Hu Q, Lu TG, Cheng ZK (2012). The role of rice HEI10 in the formation of meiotic crossovers. PLoS Genet.

[CR222] Wang YX, Xiao R, Wang HF, Cheng ZH, Li WX, Zhu GF, Wang Y, Ma H (2014). The *Arabidopsis*
*RAD51* paralogs *RAD51B, RAD51D* and *XRCC2* play partially redundant roles in somatic DNA repair and gene regulation. New Phytol.

[CR223] Wang X, Lu J, Lao K, Wang S, Mo X, Xu X, Chen X, Mo B (2019). Increasing the efficiency of CRISPR/Cas9-based gene editing by suppressing RNAi in plants. Sci China Life Sci.

[CR224] Wang Y, Dong Z, Ma Y, Zheng Y, Huang S, Yang X (2023). Comprehensive dissection of meiotic DNA double-strand breaks and crossovers in cucumber. Plant Physiol.

[CR225] Waterworth WM, Altun C, Armstrong SJ, Roberts N, Dean PJ, Young K, Weil CF, Bray CM, West CE (2007). NBS1 is involved in DNA repair and plays a synergistic role with ATM in mediating meiotic homologous recombination in plants. Plant J.

[CR226] Williams RS, Williams JS, Tainer JA (2007). Mre11-Rad50-Nbs1 is a keystone complex connecting DNA repair machinery, double-strand break signaling, and the chromatin template. Biochem Cell Biol.

[CR227] Wold MS (1997). Replication protein A: a heterotrimeric, single-stranded DNA-binding protein required for eukaryotic DNA metabolism. Annu Rev Biochem.

[CR228] Wu TC, Lichten M (1994). Meiosis-induced double-strand break sites determined by yeast chromatin structure. Science.

[CR229] Wyatt HDM, West SC (2014). Holliday junction resolvases. Cold Spring Harb Perspect Biol.

[CR230] Xu LH, Kleckner N (1995). Sequence non-specific double-strand breaks and interhomolog interactions prior to double-strand break formation at a meiotic recombination hot spot in yeast. EMBO J.

[CR231] Xue ZH, Li YF, Zhang L, Shi WQ, Zhang C, Feng MS, Zhang FF, Tang D, Yu HX, Gu MH, Cheng ZK (2016). OsMTOPVIB promotes meiotic DNA double-strand break formation in rice. Mol Plant.

[CR232] Yao H, Zhou Q, Li J, Smith H, Yandeau M, Nikolau BJ, Schnable PS (2002). Molecular characterization of meiotic recombination across the 140-kb multigenic *a1-sh2* interval of maize. P Natl Acad Sci USA.

[CR233] Yokoo R, Zawadzki KA, Nabeshima K, Drake M, Arur S, Villeneuve AM (2012). COSA-1 reveals robust homeostasis and separable licensing and reinforcement steps governing meiotic crossovers. Cell.

[CR234] Yu HX, Wang M, Tang D, Wang KJ, Chen FL, Gong ZY, Gu MH, Cheng ZK (2010). OsSPO11-1 is essential for both homologous chromosome pairing and crossover formation in rice. Chromosoma.

[CR235] Zelkowski M, Olson MA, Wang MH, Pawlowski W (2019). Diversity and determinants of meiotic recombination landscapes. Trends Genet.

[CR236] Zhang LS, Ma H (2012). Complex evolutionary history and diverse domain organization of SET proteins suggest divergent regulatory interactions. New Phytol.

[CR237] Zhang C, Song Y, Cheng ZH, Wang YX, Zhu J, Ma H, Xu L, Yang ZN (2012). The *Arabidopsis thaliana* DSB formation (AtDFO) gene is required for meiotic double-strand break formation. Plant J.

[CR238] Zhou SR, Wang Y, Li WC, Zhao ZG, Ren YL, Wang Y, Gu SH, Lin QB, Wang D, Jiang L, Su N, Zhang X, Liu LL, Cheng ZJ, Lei CL, Wang JL, Guo XP, Wu FQ, Ikehashi H, Wang HY, Wan JM (2011). *Pollen Semi-Sterility1* encodes a Kinesin-1-Like protein important for male meiosis, anther dehiscence, and fertility in rice. Plant Cell.

[CR239] Zickler D, Kleckner N (2015). Recombination, pairing, and synapsis of homologs during meiosis. Cold Spring Harb Perspect Biol.

